# “… the way we welcome them is how we will lead them to love family planning.”: family planning providers in Rwanda foster compassionate relationships with clients despite workplace challenges

**DOI:** 10.1186/s12913-021-06282-x

**Published:** 2021-04-01

**Authors:** Hilary M. Schwandt, Angel Boulware, Julia Corey, Ana Herrera, Ethan Hudler, Claudette Imbabazi, Ilia King, Jessica Linus, Innocent Manzi, Madelyn Merritt, Lyn Mezier, Abigail Miller, Haley Morris, Dieudonne Musemakweli, Uwase Musekura, Divine Mutuyimana, Chimene Ntakarutimana, Nirali Patel, Adriana Scanteianu, Biganette-Evidente Shemeza, Giànna Sterling-Donaldson, Chantal Umutoni, Lyse Uwera, Madeleine Zeiler, Seth Feinberg

**Affiliations:** 1 Western Washington University, 516 High Street MS9118, Bellingham, WA 98225 USA; 2 Spelman College, 350 Spelman Ln SW, Atlanta, GA 30314 USA; 3 Wheaton College, 26 E Main St, Norton, MA 02766 USA; 4 Northwest Vista Community College, 3535 N Ellison Dr., San Antonio, TX 78251 USA; 5 Whatcom Community College, 237 W Kellogg Rd, Bellingham, WA 98226 USA; 6 INES, Ruhengeri, Musanze Rwanda; 7 Xavier University, 1 Drexel Dr, New Orleans, LA 70125 USA; 8 UMBC, 1000 Hilltop Cir, Baltimore, MD 21250 USA; 9 SUNY Oswego, 7060 NY-104, Oswego, NY 13126 USA; 10 Western Oregon University, 345 Monmouth Ave N, Monmouth, OR 97361 USA; 11 Eastern Oregon University, One University Blvd, La Grande, OR 97850 USA; 12 University of Kentucky, Lexington, KY 40506 USA; 13 Arcadia University, 450 S Easton Rd, Glenside, PA 19038 USA; 14 Rutgers, New Brunswick, NJ USA; 15 Drexel University, 3141 Chestnut St, Philadelphia, PA 19104 USA

## Abstract

**Background:**

Rwanda has markedly increased the nation’s contraceptive use in a short period of time, tripling contraceptive prevalence in just 5 years between 2005 and 2010. An integral aspect of family planning programs is the interactions between family planning providers and clients. This study aims to understand the client-provider relationship in the Rwandan family planning program and to also examine barriers to those relationships.

**Methods:**

This qualitative study in Rwanda utilized convenience sampling to include eight focus group discussions with family planning providers, both family planning nurses and community health workers, as well as in-depth interviews with 32 experienced modern contraceptive users. Study participants were drawn from the two districts in Rwanda with the highest and lowest modern contraceptive rates, Musanze and Nyamasheke, respectively Data analysis was guided by the thematic content approach, Atlas.ti 8 was utilized for coding the transcripts and collating the coding results, and Microsoft Excel for analyzing the data within code.

**Results:**

Data analysis revealed that, despite workplace related challenges – including inadequate staffing, training, and resources, relationships between providers and clients are strong. Family planning providers work hard to understand, learn from, and support clients in their initiation and sustained use of contraceptives.

**Conclusion:**

Given the existing context of purposeful efforts on the part of family planning providers to build relationships with their clients, if the current level of government support for family planning service provision is enhanced, Rwanda will likely sustain many current users of contraception and engage even more Rwandans in contraceptive services in the future.

## Background

Analysis suggests that if all maternal and child health needs were met – the health and life impacts would be significant: maternal mortality would decline by 70%, newborn deaths would reduce by 40%, there would be two thirds fewer unintended pregnancies, and unsafe abortions would drop by 73% [1]. Family planning, a crucial maternal health service, contributes to reductions in maternal, infant, and childhood mortality via prevention of unwanted pregnancies and a reduction in births [2]. The impact of family planning is larger than the individual, as family planning use aids families, local communities, and entire nations in achieving better health outcomes [3]. While there is recognition that strong health systems positively impact health, there is less consensus on how to strengthen them [4].

An integral aspect of a strong health system are the people who provide the services. Health providers everywhere face challenges – but those in developing nations often face more substantial barriers to health service provision than providers from developed nations. Health providers from developing nations commonly note challenges with inadequate staffing, training, and supplies [5–8].

When faced with these challenges, it is difficult for health providers to offer the health services they are trained to deliver, such as the abusive treatment of clients by providers during childbirth [9, 10]. Against this backdrop of lower quality care during childbirth, what about family planning care provision by health workers? An important piece of family planning programs is the interactions between family planning providers and family planning clients [11], as identified by Bruce and Jain in their pivotal work on quality in family planning programs [12, 13], and later revised in the context of rights-based care [14]. Within these frameworks, there are a number of elements related to quality, one of which is interpersonal relationships. With interpersonal relationships, the emphasis is on treating the client as a partner in the contraceptive use journey with dignity and respect.

Rwanda has markedly increased its contraceptive prevalence rate in a short period of time, tripling contraceptive use among married women from 12 to 52% in just 5 years, between 2005 to 2010, with use nominally rising 5 years later to 53% in 2015 [15]. Rwanda is a stand-out in terms of family planning program success [16, 17]. Amidst this backdrop of rapid increase in contraceptive use in Rwanda, this study aims to explore the potential role of one contribution among many to the rapid increase in contraceptive use in Rwanda – the interpersonal relationships between family planning providers and clients, and any workplace challenges that may negatively impact provider’s ability to foster those relationships.

## Methods

This qualitative study was conducted from February to July of 2018. Qualitative data were collected via focus group discussions (FGD) and in-depth interviews (IDI) in the Musanze and Nyamasheke districts of Rwanda (the areas of the country with the highest (67%) and lowest (34%) rates of modern contraceptive prevalence rates, respectively [15]). In total, eight FGDs with 88 family planning providers (four FGDs with family planning nurses and four FGDs with community health workers) and 32 IDIs with experienced modern contraceptive users were conducted, split evenly by district. The study design included eight FGDs to meet the goal of having two FGDs per study characteristic – in this case, geographic location and provider type, and 32 IDIs to have at least eight IDIs per characteristic, geographic location and contraceptive user type.

All study participants were sampled using convenience sampling. Governmental and non-governmental employed Rwandans who knew the family planning providers in each district recruited the family planning providers who participated in this study. The inclusion criteria for the family planning providers was that the family planning nurses and CHWs work primarily in family planning.

The experienced contraceptive users were recruited via family planning providers. All potential study subjects were recruited face-to-face or from telephone calls. The study design initially called for eight study participants in each district who were at least 18 years of age and current modern contraceptive users for at least 6 months, and another eight participants who were at least 18 years of age and had discontinued a modern contraceptive method in the last 6 months. After recruitment and data collection had already begun, it became clear that there was a misunderstanding between the researchers and the recruiters in terms of what constituted discontinuation of modern contraception – as well as difficulty in finding recent discontinuers. As a result, the contraceptive users included in this study were all experienced modern contraceptive users, and the vast majority were currently using modern contraception.

Six data collectors, two male and four female university educated students, who had no previous qualitative data collection experience, received a week long intensive training workshop that included review of research ethics, consenting study participants, reviewing the consent forms, qualitative data collection techniques, review of the topic guide, and ample practice using the topic guide in training scenarios. The topic guides were developed based upon prior experience conducting qualitative research in Rwanda, and other nations, on the topic of family planning. No changes were made to the topic guide during data collection.

Each focus group discussion and interview was conducted in Kinyarwanda. Each study participant participated in the study at one time point only. The FGDs took place inside private rooms at public institutions. The IDIs took place in outdoor areas selected by the interviewer and participant for their privacy. Only data collectors (two for the FGDs, and one for the IDIs) and the study participants were present during data collection. Data collectors and study participants did not know each other prior to data collection so basic introductions were made immediately prior to consent and data collection. The impetus for the study was clear through the reading and discussion about the consent form. There were no respondents who refused to participate in the study.

Audio recordings and field notes were transcribed into English by the data collectors working alongside native English speakers. All transcription occurred in Rwanda. Transcripts were not returned to participants for comment. The FGDs lasted on average 2 hours, and the IDIs lasted 43 min.

The entire research team participated in coding the data. Each researcher coded the same transcript individually. Then the research team met as a group to share coding results and agree on a master code list and definitions. All codes were generated in-vivo, from the data. Then the researchers coded the remaining transcripts. Data analysis was guided by the thematic content analysis approach [18] using inductive thematic saturation [19] and executed using Atlas.ti 8 software [20] and group level matrices in Microsoft Excel.

Approval from the Institutional Review Boards at Western Washington University in Bellingham, Washington and the Rwandan Ministry of Education in Kigali occurred prior to data collection. Study participants were compensated 10,000 Rwandan francs (~$10 USD) for their time and any transportation costs.

To address issues of validity and reliability during data collection, analysis, and interpretation the research team employed data, method, and investigator triangulation, as well as low inference verbatim descriptions to illustrate the study themes [21, 22].

## Results

### Socio-demographic characteristics

The family planning nurses were on average 40 years old, CHWs 47, and experienced contraceptive users were 38. Experienced contraceptive users in this study had on average four children, while CHWs had five and nurses had three. All of the experienced contraceptive users were female and most of the nurses were, but just about half of the CHWs in this study were female. The most common method used among experienced contraceptive users and CHWs was the injectable (~ 40%) while it was the implant among family planning nurses (55%) (see Table 1).

**Table 1:** Sociodemographic Characteristics of Family Planning Providers and Experienced Contraceptive Users, Rwanda 2019

	FP Nurse	CHW	Experienced User
	(*n* = 40)	(*n* = 48)	(*n* = 32)
Age, mean (SD)	40.3 (6.7)	46.8 (7.1)	37.7 (6.0)
Parity, mean (SD)	2.9 (1.3)	4.6 (1.5)	3.9 (1.8)
Sex, % female	95	54	100
Contraception Used, %	90	90	100
Condom	3	15	22
Pill	0	21	13
Injectable	18	42	41
Implant	55	17	19
IUD	13	0	0
Sterilzation	8	17	4

In Rwanda, family planning nurses and community health workers (CHWs) partner to provide family planning services to members of their communities. Respondents revealed a number of challenges that create barriers in their work to deliver services, while simultaneously outlining a pattern of success via compassionate counseling of new and continuing family planning clients.

### Provider challenges

Providers noted that the work they do is not without challenge and sacrifice, including both professional and personal obstacles.

We face a lot of challenges. But because we give ourselves out to the community, and the courage that we have, and the friendship we have in the village - we have to do it.

CHW, 44 years, female, Musanze

This job of ours is to work, to sacrifice, teach what’s possible, today you sit here … tomorrow there, until in the end they will be able to understand.

CHW, 44 years, male, Musanze

Family planning providers were aware that the challenges in their jobs threatened their ability to have successful relationships with clients. More providers contributed to themes regarding the challenges they faced, although clients contributed to some of those themes as well. The challenges providers confronted fell into the following subthemes: human resource shortages, insufficient supplies, and desire for more training. There were two additional themes that only arose as challenges for CHWs - threats to personal time and a desire for financial remuneration.

### Human resource shortages

The most common challenge providers noted was a need for additional personnel. According to respondents, adequate staffing would alleviate the majority of provider’s concerns.

M: In your everyday job are there challenges that you face? R: The biggest challenge is that we have few employees.

Nurse, 42 years, female, Musanze

I: What is the worst problem you have faced in family planning? R: The problem I experienced is that we don’t have sufficient family planning providers in the village, and also we don’t have more nurses or doctors in the domain of family planning program.

Implant user, 35 years, four children, Nyamasheke

Family planning providers noted how emergency services take precedence over preventative services in terms of staffing, which then impacts waiting times for new and returning clients. The main concern about the lack of adequate staffing was the delay in service delivery, due to competing priorities for nurses at facilities, leading to long wait times for the family planning clients as well as the lack of services available.

She may potentially come to the clinic and we know that she has an appointment; however, due to the limited amount of nurses she may find that we will prioritize emergency situations before dealing with her … When she grows impatient she will return home without assistance.

Nurse, 40 years, male, Musanze

Some people are accustomed to their CHW, so if they go to the health center it can take a long time for them to be treated. It can take about 3 hours before she will get treated. So she thinks she is wasting her time, but the problem is that there are many people waiting for the same service. She can feel like she is being treated unfairly. The people in the community think that this is a challenge, and can think that going to the health center is a waste of time.

CHW, 43 years, female, Musanze

… advice I can give is that there are times where you can go to the health center when you want to get family planning services but face the problem that you find that the nurses that are in charge of family planning, they are doing other emergency services. So I can recommend that we can have one permanent family planning services provider at the health center that works every day …

Injectable user, 43 years, four children, Nyamasheke

The shortage of needed staff also led to inadequate service provision-which negatively impacts the women using contraception.

She may come once for injection, and you complete her file, you discuss with her the main points, but you give the brief explanation because you don’t have time to go deeply …

Nurse, 45 years, female, Musanze

In order to handle the shortage of human resources at some facilities – family planning services are restricted to a few days a week; however, the schedule of these services may not be known to all who want to access the services.

We want family planning service to be allowed to work every day by the health center just like the other services they provide not just for Tuesday and Friday. For example, due to only 2 days of services a woman can risk becoming pregnant if she had wanted to see someone sooner. I believe it should be open weekly to be easier for women to come in whenever.

CHW, 39 years, female, Nyamasheke

… add more staff, but also to have an employee who is solely in charge of family planning. So that in the case that a woman comes in for family planning or contraceptives they will know for sure that they will find a doctor who is in charge of that service and there all the time.

Nurse, female, Musanze

Primary concerns and critiques included a need for additional CHWs in the villages. While some respondents said the amount of CHWs was sufficient, in both Musanze and Nyamasheke more identified a need for additional access to more staff.

… to add more community health workers in the village because you see that we have too many villagers but they only have three community health workers; so that they can help a woman to be comfortable because if the community health workers are few they can’t reach all the women, but if they can increase the number of community health workers to five or to six they can reach more women …

Condom user, 41 years, five children, Musanze

### Insufficient supplies

Nurses provided suggestions regarding supplies – to have all of the necessary equipment on hand to serve the clients, transportation for those who are coming to the clinic – or to follow up with those that did come to the clinic, and better methods of communication between the providers and the clients.

CHWs discussed additional ways that the Rwandan government could support them with resources – also through adequate contraceptive supplies. Presumably this includes contraceptive methods themselves, as well as the supplies required for administering and keeping track of the supplies. Storage of supplies was also noted as a common request. Resource comments were noted six times more often by Nyamasheke CHWs as compared to Musanze CHWs.

It would also be beneficial if they gave CHWs enough material because it is hard when the client comes looking for a contraceptive method for the first time and the materials needed are not available.

CHW, 45 years, female, Nyamasheke

The majority of responses from providers regarding a lack of resources shared a concern for the impact it would have on current and future family planning clients. Providers recognized that negative family planning experiences for clients could have repercussions for other women in the community and their desire to seek out family planning services.

### Desire for more training

A common need for more training of providers also emerged from the data analysis – both in general family planning services and for more specific technical needs, i.e., providers trained in the provision of long term and permanent methods.

The other thing they can help with … you see not all the staff are trained about family planning. For example, a person can come to the hospital and need to use the IUD method and has a problem, because there is only one person who is trained about IUDs. And that person who is trained about IUDs is not always at the health center. So if there is a person who needs that method, they have to wait. This is a problem.

Nurse, 35 years, female, Nyamasheke

The topic of training CHWs also arose in nearly all FGDs and a quarter of the IDIs.

… they have to have a lot of information about contraceptives because they cannot give what they don’t have.

Pill user, 45 years, two children, Nyamasheke

There was a recognition of the need for ongoing training of CHWs – as well as the impact of training CHWs on a broader audience of the entire community.

I think that they should continue to give more training because we will benefit from them.

Injectable user, 41 years, five children, Musanze

### Threats to personal time

CHWs in every focus group brought up issues related to the time it takes to serve their neighbors as a CHW.

Another problem we face is that to do well in your work, you need to have a lot of time to put into it.

CHW, 46 years, male, Musanze

CHWs worked hard to follow-up with neighbors.

… some of the mothers might have appointments to come see us at our house but they won’t show up and as a CHW you are worried because they did not show and then you feel like you must go out and visit them or find them. That problem keeps following you, you get to her house and find she is not there and that means you have to go back. A CHW faces a lot of challenges and sometimes you have to go and look for people more than three times. You feel you have to make the sacrifice and not stop until you find them.

CHW, 56 years, male, Musanze

CHWs noted how the time it takes to serve clients takes away from their own needs to take care of their families either through caretaking or for financial gain.

I use most of my time as a CHW instead of caring for my family.

CHW, 46 years, female, Nyamasheke

Because I have a lot of clients at my house, my kids eat late or have to sleep without food. To be a CHW means sacrifice.

CHW, 44 years, female, Musanze

Even if I am in the middle of my own job, when the client comes I must help her without taking care of my own tasks that could bring in profit.

CHW, 51 years, female, Nyamasheke

### Desire for financial remuneration

The topic of compensation for hard work arose in every FGD with CHWs and even among the IDIs with experienced contraceptive users. CHWs felt that “motivation” in terms of financial payment would help them accommodate their own needs to provide for their families while maintaining their ability to serve their neighbors. CHWs also noted how their work would feel more valued and appreciated with financial compensation. This topic arose three times more often among Nyamasheke CHWs as compared to CHWs in Musanze.

A difficulty we encounter is we use all of our time as CHWs and do not work for our families. We do all of this without financial compensation.

CHW, 51 years, female, Nyamasheke

According to the time we spend doing this, we deserve to be compensated, even if it is not a big sum. We need to get that support. This will help me. For example, suppose that I am a farmer, if the government gives me some money, I can send someone else in my place to cultivate my field while I am doing this work as a CHW.

CHW, 46 years, male, Musanze

...our community health workers also need to work for their families and sometimes they cannot manage to both provide for their families and also to work for us when we are seeking their services. Because sometimes we go to look for community health workers and find that they are not available due to maybe their personal plans. And based on this, I think that the government of Rwanda may look for ways to give community health workers motivation and money also so that they can work as if it is their job. So that any time, hour to hour, we can access them.

Injectable user, 32 years, three children, Musanze

### Rewarding provider-client relationships

Despite the numerous challenges that family planning providers face in contraceptive service provision in Rwanda, there was more emphasis on the positive in their loving and compassionate interactions with their communities. The nurses and CHWs describe their interactions with family planning clients with strong senses of empathy, respect, and the importance of utilizing listening skills. Providers came across as patient, kind, and loving with their clients.

It’s inside you to give every client the services she wants.

Nurse, 44 years, female, Nyamasheke

… welcome people well, because the way we welcome them is how we will lead them to love family planning.

Nurse, 38 years, female, Nyamasheke

In terms of provider and client relationships, many positives were reported, primarily by providers, but also clients. The positive subthemes included: reliable access to providers and methods, providing comprehensive information, respecting client privacy, compassionate counseling, and counseling dissatisfied users with care.

### Reliable access to providers and methods

Many study participants noted that they did not find any barriers in accessing family planning providers and contraceptive methods. Of those who mentioned a lack of barriers, more lived in Musanze than in Nyamasheke.

The other thing I can say the country helped us in is that at the health center every nurse is capable to give family planning methods. This means that they trained us about family planning. Every hospital staff knows how to give family planning methods.

Nurse, 35 years, female, Nyamasheke

Nothing is difficult to us in getting services because anytime you go to the health center or to the community health workers they try to help us every day so it is also easier for us to get medicine.

Implant user, 36 years, three children, Nyamasheke

They are already ready to help us. Even if you come on Sunday they help you.

Condom user, 38 years, two children, Nyamasheke

I: Is there a time you wanted to get the injectable, but it was not available?

R: No, every time I want it I can get it.

Injectable user, 41 years, six children, Musanze

Despite the ease of access with community health workers, a few women in Nyamasheke voiced their preference for nurses at the health center due to their superior knowledge. Regardless of the fact that accessing nurses is often more challenging in terms of scheduling and travel distance. Nurses are viewed as more knowledgeable about family planning and broader health concerns as well.

For me, I don’t like to get the method from the community health workers. When I want the method I go to the health center and I go to look for nurses, because the community health workers know many things but there are things they don’t know. There are things you can ask them and they won’t have more information about. That’s why I choose to go to the health center.

Pill user, 45 years, two children, Nyamasheke

### Providing comprehensive information

Providers shared the all-inclusive information they would provide to the clients – it was clear they wanted the clients to fully understand their options, so they could make informed decisions. When clients did decide on a method, providers wanted to help them strategize how to make the experience successful and sustainable.

… I will show them the good and the bad of using family planning.

Nurse, 49 years, female, Nyamasheke

### Respecting client privacy

Providers noted how they would treat clients with respect – and honor their privacy.

… she will come to you according to how you present yourself. We emphasize a lot that we will keep their secret if they come to us.

CHW, 45 years, female, Musanze

The CHW that understands her circumstances will help her to get an appointment with family planning providers in order to keep the services she gets a secret. CHWs are going to protect this secret so that no one can find out that she is using contraception.

CHW, 61 years, male, Nyamasheke

It was particularly clear that CHWs would maintain client confidentiality – as a way to protect their clients but also to avoid ruining current and future client relationships.

… a CHW has to be a closet of secrets … if you are not a closet of secrets your advice is already broken.

CHW, 56 years, male, Musanze

### Compassionate counseling

Nurses were confident in their ability to provide and treat clients with respect – and that the outcome of their efforts would be positive.

If the discussion with her was successful, there is no other thing that can stop a woman from using a contraceptive method.

Nurse, female, Musanze

Similarly, the vast majority of women reported positive experiences with providers and felt confident in conversing with them regarding a variety of issues. Providers were reported to be knowledgeable, helpful, and supportive of women’s decisions regarding family planning use. Musanze participants contributed to this theme more so than Nyamasheke contraceptive users.

I: How did they receive you at the health center?

R: They received me well, with a smile and with much appreciation, and they give more advice about the advantages of using family planning.

Injectable user, 31 years, three children, Musanze

When contraceptive users were asked what family planning providers could do to improve most responded that they could not give advice because they perceived providers as already doing all that they could.

I have never had a problem with the services given by family planning providers so I don’t have any advice for them because they do a great job and I am thankful to them.

Injectable user, 26 years, one child, Musanze

It is difficult for me to give advice for family planning providers because for me now I see the way that they provide services to us is good, they receive us well and they always take care of us …

Pill user, 34 years, two children, Musanze

### Counseling dissatisfied clients with care

Providers also shared the compassionate way they receive and counsel current dissatisfied family planning users.

I need to have a deep conversation with her, because I need to know everything and after we can make a decision based on our conversation.

Nurse, 29 years, female, Nyamasheke

… they will discuss why she stopped using the pill in those 2 months and if she wants to continue using family planning. They will work together in order to find another method for her to use.

Nurse, 34 years, female, Musanze

A common theme that arose was providers counseling unsatisfied users with the experience of side effects. These conversations were handled with great sensitivity.

She might start the method of taking pills. And then a few days later, she will gain weight, she will be just like my size. And she might not be able to handle it and she might come back saying she wants to stop. At that moment we will have another discussion, but different from the first one … You have to remind her of the first discussion you had about side-effects that she is going to have. And then you will ask her, between getting fat or getting pregnant, what would be more of a burden?

Nurse, 50 years, female, Nyamasheke

The positive influence of providers likely plays a strong role in the continued use of contraception despite experienced side effects.

## Discussion

### Provider challenge: human resource shortages

Family planning providers and clients both noted workplace challenges health care workers face in their desire to provide clients with quality family planning services. The most common concern that both providers and clients noted was insufficient human resources – at both the health center and community levels. This theme has been noted in Rwanda [23, 24] and other contexts as well [6, 7, 25]. Interestingly, nurses at the health facilities seemed sensitive to the fact that wait times at facilities were more highly scrutinized since introduction of the CHW program and the ensuing client’s easier access to family planning services in the community – likely due to an awareness of wait times on client satisfaction with service delivery [25].

### Provider challenge: insufficient supplies

Providers also brought up concerns about inadequate supplies. While family planning nurse supply concerns revolved around opportunities to enhance engagement with clients, particularly with follow-up, which has been noted as an essential aspect of quality of care [11, 14, 26, 27] – CHWs concerns mostly centered on logistical needs, a concern also linked to impacts on quality of care [6, 8, 25]. Notably, among CHWS, these concerns were brought up much more often in Nyamasheke as compared to Musanze.

### Provider challenge: desire more training

Additionally, concerns about adequate training arose – a concern found in other contexts as well [5, 6, 8]. Family planning nurses desired more method specific training, particularly for long-acting and reversible methods [6, 8]. CHWs and community members were particularly interested in CHWs having ongoing training – as they recognized the reach of CHW education on entire communities. Research shows that perceptions of provider competence impact client’s satisfaction with family planning services [25].

### CHW provider challenge: threats to personal time & desire for financial renumeration

Challenges specific to CHWs included the sacrifice of time to meet the needs of their community members, often interfering with their ability to meet the needs of themselves and their families, and this issue was correlated to concerns about the lack of financial remuneration. The Rwandan Government has acknowledged these challenges for their CHW program in national documents [28], as well as in other research [29]. CHWs and family planning users were aware of the impact of clients on CHWs personal lives – in terms of time and profit – and understood that if CHWs were financially compensated for their efforts the impact would be felt by the entire community.

### Recommendations for the identified workplace challenges

The study participants provided potential solutions to the human resource shortages, all of which included hiring additional staff, such as: offer family planning every day of the week at health facilities, hire health facility staff dedicated to solely providing family planning services, and increase the numbers of CHWs per village from three to five or six, recommendations echoed in other research on client satisfaction [25].

In order to address the issue of inadequate supplies there is a need for the Rwandan Government to allocate greater funding to the family planning program so there are adequate funds for the supplies needed for family planning nurses and CHWs. Special attention should be paid to allocating resources for nurses and CHWs in Nyamasheke, to execute their jobs, as these areas impact quality of care.

The Rwandan Government, and others, should allocate more funds to nurses for long-acting method training to reduce client barriers to the same. In addition, ongoing training for new and continuing CHWs should be prioritized as the CHWs are an important source of family planning information for many Rwandans.

The Rwandan Government should continue to find ways to compensate CHWs for their time and efforts – as the compensation will alleviate both of the challenges noted specific to CHWs in this study, a challenge recognized by the Government of Rwanda [28] and others [29]. Some have cautioned that renumeration of social goods is better than cash with CHW programs [30].

### Rewarding client-provider relationships

Despite the workplace related challenges, Rwandan family planning providers in this study describe their interactions with community members and clients as one would who shares a deep concern and care for those they serve. Building relationships with clients based around both empowerment and empathy, providers share how they listen to and work collaboratively with them to find solutions to their problems, an important aspect of quality of care [14]. Research has shown that when providers treat clients humanely, and develop a relationship with them, clients are more likely to seek out health providers in the future [31], initiating and continuing contraceptive use [7, 32–37], as well as selecting longer term methods contraceptive use [26]. Providers, and clients, both noted the importance of reliable access to providers and methods. While this also arose as a challenge – more noted access as reliable than not. Reliable access to providers is key to client satisfaction [25]. A few clients in Nyamasheke did voice a preference for nurses over CHWs due to their superior knowledge and skills, a preference noted in other contexts as well [25].

Family planning providers also noted the importance of presenting clients with all of the information – about the best parts of contraception but also the potential negative consequences, especially in terms of side effects, an important aspect of counseling best practices [25, 33]. Providers were also aware of the importance of maintaining clients’ confidentiality and the impact of their individual interactions with clients on their future relationship with the broader community. Other research has found the importance of privacy, confidentiality, and information sharing on contraceptive uptake [14, 25, 38]. It is important to note that experienced contraceptive users were less likely to go into detail about interactions with providers, perhaps due to feeling they did not have the role or authority to critique others who are experts in an area they are not, but users indicated satisfaction with the interactions they have with providers.

### Continuing to support rewarding client-provider relationships

In Rwanda, the internal motivation among family planning providers to foster positive provider-client relationships is strong – likely due to the support for family planning success by the leadership of the country [39], alongside structural support made available to providers in family planning service provision [23, 40]. Challenges identified interfere with the desire to foster and maintain these relationships – particularly related to insufficient human resources, training, time, and financial remuneration. Given the positive relationships established, and the desire to continue to develop these types of relationship through collaborative client-provider interactions, it is in the best interest of the Rwandan government to continue to remove barriers to offering and maintaining these relationships in order to sustain existing support and to build on the successes from the last decade.

This study has a number of strengths. The research included both experienced modern contraceptive users as well as family planning providers – health facility nurses and CHWs, allowing for triangulation of the data findings. The data were also collected in two different districts, purposively selected for having the highest and lowest modern CPR in the nation. Despite the strengths, the study does also have some limitations. Misunderstanding between the study participant recruiters and the researchers led to the modern contraceptive user sample not including discontinued users. The data collectors did not have previous experience with qualitative data collection. Transcription and translation occurred in a single step, instead of two, reducing the accuracy of translation. Finally, family planning providers recruited the experienced contraceptive users – so those recruited likely have a more positive experience with the family planning program in Rwanda.

The use of family planning is vitally important to individuals and couples in order to prepare for and execute the plan for the timing and number of births – as well as communities and nations to have stable population growth for adequate resources and financial opportunities for all persons. Efforts by individuals, providers, and governments to facilitate effective family planning programs are essential to create this enabling environment. While Rwanda has made impressive strides in the family planning program, work remains to increase access to and use of contraception by even more Rwandans, as well as continuing to meet the needs of current users. Attention to what is working well and continuing to support those successes – as well as removing barriers to continuing the good work of family planning providers, is likely to have ramifications beyond the individuals impacted, as these study participants sagely elucidate.

## Data Availability

The transcripts analyzed for the current study are not publicly available due to ethical reasons, the sensitivity of the information, but are available from Western Washington University’s Research Compliance Office, Janai Symons, at lanej4@wwu.edu upon reasonable request.
